# Evaluation results of using GAP II acetabular cage for acetabulum in revision total hip arthroplasty

**DOI:** 10.1097/MD.0000000000032056

**Published:** 2022-11-25

**Authors:** Afshin Taheriazam, Amin Saeidinia

**Affiliations:** a Department of Orthopedics Surgery, Tehran Medical Sciences Branch, Islamic Azad University, Tehran, Iran; b Mashhad University of Medical Sciences, Mashhad, Iran.

**Keywords:** acetabular cage, GAP II, hip arthroplasty, reconstructive surgery, revision arthroplasty

## Abstract

Acetabular revision arthroplasty with major bone loss is one of the most difficult operations in hip arthroplasty, The graft augmentation prosthesis (GAP) has been designed particularly as an implant for revision acetabular reconstruction. We evaluated the use of GAP II acetabular cage in revision of acetabulum in total hip arthroplasty. From 2009 to 2014, we performed revision total hip arthroplasty in patients with acetabular defects by cage (GAP II) in patients referred to Milad and Erfan Hospitals, Tehran, Iran. We included all patients in class 3a and 3b of Paprosky bone loss classification and type III bone loss according to the system of the American Academy of Orthopedic Surgeons. We used SPSS software Ver 19 and descriptive tests, Chi square and independent *t*-test were used for analysis. There were 221 men (71.99%) and 86 women (28.01%) with an average age of 51.3 ± 21.7 years (range, 35–86 years). The Modified Harris Hip Score (MHHS) improved significantly at the last follow-up compared with the preoperative MHHS (*P* < .001). The mean MHHS was 40 (range, 29–44) preoperatively and 92 (range, 86–95) at the last follow-up. There were no major intraoperative complications during acetabular reconstruction. Our findings showed that using GAP II acetabular cage in the restoration of acetabulum in hip revision surgery is significantly desirable.

## 1. Introduction

The implant that is used in total hip replacement contains 3 parts as follows; basic (stem - fits into the femur), balls (ball - replace the spherical head of the femur), and socket (cup - to replace the defective pelvic cavity). It is worth mentioning that in order to match the implant with different bodies, each part of the implant designs in different sizes. Additionally, the type of implant to be used depends on the age, weight, activity level, and health and the patient’s bone density.^[1]^ However, today, the base part of the most of the implants are made out of titanium alloys, chromium or cobalt-chrome which has a porous and leaky surface, allowing bone in-grow into it. The features of these implants that can be mentioned are corrosion resistance, biocompatibility, wear resistance, mechanical properties similar to the natural joint and having the necessary and sufficient standards. Total hip arthroplasty (THA) with cement is performed in 3 ways. In the first method, cement is used to secure the implant. Thus, the femoral canal will be filled with cement and then implant will be inserted, after a while it will be fixed in its place.^[2–5]^ These patients can soon put their weight on the member and with a physiotherapy session (almost immediately after the surgery) walk without any help.

The most common reason for failure of THA is periprosthetic osteolysis and loosening of hip implants.^[6]^ The rate of osteolysis varies between femoral and acetabular sides, and it is more common on the acetabular side. This is why acetabular cup loosening is the main cause for revision in long-term studies.^[7]^ This loosening usually is associated with pain, reduced function, and instability of the implant. There are 2 main problems to solve when an orthopedic surgeon has to approach acetabular revision. First of all, even with bone loss due to the loosening of the previous implant, obtain primary fixation of the new prosthesis; then reach postoperative implant stability. This second issue could be more difficult when isolated acetabular revision is performed.^[8,9]^

To address this risk, industries and surgeons have developed a variety of surgical hardware and strategies such as jumbo femoral heads, constrained acetabular liners, and dual-mobility cup.^[10]^ Another way to face these problems is, in order to obtain fixation, the implantation of a shell in the better position allowed by the bone defect; then obtain stability cementing a polyethylene liner in the shell with a partially independent version and verticality.^[10]^Total hip replacement with cement for elderly patients, patients with rheumatoid arthritis or young patients with low bone density is recommended^[11]^ because these patients are less likely to put pressure on the cement to the extent that a breakage happen. The second method is to replace the hip without cement. In 1980, an implants were designed which could directly be connected to the bone without cement. There was a coating on the surface of these implant, which have tiny holes in the tissue areas that makes it possible to provide increased bone absorption. The severity of these implants’ stability is largely based on the growth of new bone, therefore the treatment duration for these patients are longer. The third method which is a combination of the mentioned methods, was introduced in 1980s. The GAP repair collections (prosthesis for strengthening grafts) in acetabular reconstruction implants are specifically designed to be a comprehensive solution to the challenges of reconstruction of acetabulum.^[12]^ The GAP II acetabular cup system, addresses the construction of acetabular with a graft bite through immobilization and proper pressure. GAP ring is a reinforced titanium ring used to reconstruct acetabular for the cases. GAP II is the second generation of acetabular reconstruction cage which has characteristics such as titanium shell molded with lower hook, upper plate, and several screws hole clusters to fix the ileum and Ischium.^[13]^ Acetabular revision cup GAP II has been designed to achieve stability in acetabular that is in a highly deficient bone condition. Each GAP device is compressed by the host bone and allograft bit or supported by structural allograft bone and is fixed in place with bone screws, at the end all kinds of learners will be cemented in their place. This allows the surgeon to achieve maximum stability.^[12]^ The current study evaluates the results of utilizing GAP II acetabular cage to repair acetabulum in revision THA.

## 2. Patients and methods

From 2009 to 2014, we performed revision THA in patients with acetabular defects (due to bearing and osteolysis) by cage (graft augmentation prosthesis (GAP) II) rings in patients referred to Milad and Erfan Hospitals, Tehran, Iran. We included all patients in class 3a and 3b of Paprosky bone loss classification^[14]^ and type III bone loss according to the system of the American Academy of Orthopedic Surgeons.^[13]^ Exclusion criteria was patients with higher bone defect classification and other contraindications for doing revision.

The specific reasons for use of this cage were: acetabular cemented cup osteolysis in 233 cases (75.89%), cup protrusion in 62 cases (20.19%), and cementless cup osteolysis in 12 cases (3.90%). Initial diagnosis was osteoarthritis in 274 patients (89.25%), developmental dysplasia of the hip in 21 patients (6.84%), and rheumatoid arthritis in 12 patients (3.90%).

There were 221 men (71.99%) and 86 women (28.01%) with an average age of 51.3 ± 21.7 years (range, 35–86 years). We classified acetabular deficiencies radiographically before surgery and confirmed the classification after removal of components during the procedure using the system reported by the American Academy of Orthopedic Surgeons classification.

We performed 307 revision cases for acetabular defects during the period of the study by cage GAP II in combination with impacted bone allografts for acetabular reconstruction. All patients underwent the procedure using a direct lateral approach under general anesthesia in operating room. We used in Bulky Allograft in 46 patients (14.98%), Augment in 15 patients (4.88%), Cup-Cage in 5 patients (1.62%), and in other 241 patients (78.50%) Morselised Allograft without impaction were used. Also in 12 patients that had previous cage failure by other surgeons, the senior author performed re-caging. Femoral side revision for osteolysis and loosening of femoral side, was done for 283 patients (92.18%). All patients had a GAP II implant used in their reconstruction, with a 28 or 32 mm polyethylene liner cemented into place. The outer diameter of the liners we used ranged from 48 to 54 mm, with an average of 50 mm. Preoperative prophylaxis against infection was given to all patients (cefazolin 1 g intravenously before the surgery followed by 1g 3 times daily for first day). Subcutaneous low molecular weight heparin (40 mg once daily) starting on the day of surgery was given to all patients for 14 days in addition to antiembolism stockings as prophylaxis against deep vein thrombosis (DVT). The average operative time was 146 minutes (range, 90–190 min). Early mobilization was used both to prevent DVT and to facilitate the functional recovery. Full weight-bearing was allowed from the day after surgery with walker onwards in all cases. They used walker for first 3 weeks and physiotherapy was performed for them outpatient in first week.

Complications were evaluated during follow up and hospital stay and patients were followed closely for a period of 3.89 ± 2.2 years (range: 3–5 years) for any possible complication and functional outcomes. Modified Harris Hip Score (MHHS) was used for functional outcome assessment. We collected data prospectively using an information sheet that had individual data for each patient. We evaluated patients clinically and radiographically at preoperatively, postoperatively, in the time of discharge, then 6 months and yearly postoperatively. We routinely obtained pelvic AP and lateral views radiography. Patients were monitored for complications related to their arthroplasties including infection, nerve injury, DVT, pulmonary embolism, dislocation and cup migration or loosening. Systemic complications including cardiac, gastrointestinal complications, cerebrovascular accidents, phlebitis/pulmonary embolism, and urinary tract infection were also noted.

We used descriptive statistical analyses to present mean and standard deviation of quantitative variables. The results were compared between the variables for statistical significance either by a Student’s *t*-test or a Mann–Whitney *U* test. Dichotomous variables were analyzed using a Chi-squared test or Fisher’s exact test. We determined survival using the Kaplan–Meier survival method. For all analyses, we used SPSS (SPSS 21.0 for Windows; SPSS Inc. Chicago, IL). *P*-Value less than 0.05 were considered as significant.

We considered all ethical issues for patient’s information and procedures based on ethical committee of Tehran branch of Azad University and ethical statements. Informed consent was obtained from each individual prior to surgery, and patients were fully informed of the potential benefits and complications.

## 3. Results

The MHHS improved significantly at the last follow-up compared with the preoperative MHHS (*P* < .001). The mean MHHS was 40 (range, 29–44) preoperatively and 92 (range, 86–95) at the last follow-up. There were no major intraoperative complications during acetabular reconstruction. At follow-up, there were 2 dislocations that one of them managed by cup exchange in DDH case and another one constrained linear cemented to cage. There was only one patient with deep infection needed for cage and all prosthesis removal due to uncontrolled diabetes mellitus was performed. There was only one case of sciatic nerve palsy which was treated conservatively. No patients sustained deep vein thromboses. There were no cases of pulmonary embolism or other systemic complications. Total rate of complications was 1.3%.

There was no evidence of loosening or cup migration and Heterotopic ossification in radiographic follow ups. At the final follow up 21 patients (6.84%) had moderate pain and other were pain free which was significantly improved in comparison with preoperative pain score. The mean of VAS score preoperatively was 9.2 ± 0.56 and decreased to 3.21 ± 1.59 at the last follow-up.

All of patients walked without external support at the final follow up. The survival rate of cage GAP II was determined 92% in nearly 4 years follow-up.

## 4. Discussion

The most common indications for acetabular revision include hip instability, aseptic loosening, periprosthetic osteolysis, and infection,^[15,16]^ with the acetabular component involved in more than fifty percent of revisions.^[16]^ Acetabular bone loss can be found in any of the revisions of THA and is one of the factors to be taken into account when determining treatment. There are multiple options for the treatment of these defects, each of which have their strengths and weaknesses, as all have common goals such as bone stock rebuild and provide mechanical stability with a maximum host bone contact.^[17]^ Rings and cages are indicated for use in revision THA with severe bone loss, as those described in Paprosky classification, as types II and III.^[1]^ When periprosthetic osteolysis and implant loosening are associated with pain and reduced function or when osteolysis threatens implant fixation, revision hip arthroplasty can be considered for treatment.

The need for revision THA continues to grow yearly. The reconstruction of acetabular bone defects remains a challenge to arthroplasty surgeons. Several methods have been tried to manage this including allograft reconstruction with hemispherical cup, oblong acetabular components, custom triflanged implants, trabecular metal cup with augments, or a reconstructive cage. The use of the acetabular impaction grafting in addition to a reconstructive cage has been an attractive option in treating these severe acetabular bony deficiencies. The cage is to bridge the bony defect and to protect the underlying bone graft as it incorporates restoring the pelvic bone stock which allows future revisions if necessary. We assessed here the use of GAP II cage for revision THA. Our findings showed an excellent result in short and mid-term outcomes. In line with our study, Hosny et al, revealed that the average Oxford Hip Score improved from 11.3 preoperatively to 32.2 after use of GAP II cage with impaction bone grafting to reconstruct severe acetabular defects. They also showed that the revision free survivorship of this construct was 100% at mean follow-up of 49 months. Radiological failure of the implant was reported in 3 cases without clinical outcomes.^[18]^ In their series of 12 patients (5 primary and 7 revision THA), Duffy et al^[19]^ reported an early failure of the GAP reinforcement ring. They reported mechanical failure of the implant in 5 of their 7 revision patients (71%). In 4 of those 5 patients, the cause of failure was attributed to the resorption of the bulk allograft, which they used to support the cage superiorly. They advised not to use this cage unless supported by host bone. In another study,^[13]^ despite of not relying on bulk allografts to support the GAP II cage, they observed early catastrophic mechanical failures in 37% of their cohort of 24 patients at an average follow-up of 36 months. They also reported 83% pelvic discontinuity that were reconstructed with the GAP II cage with no more hardware fixation.^[13]^ In another study, Thaler et al reported that 6 of the 64 patients were revised at a mean follow-up of 27.6 months, 2 each for implant failure, infection, and recurrent dislocation.^[20]^ Sembrano et al demonstrated that 5-year cage revision free survivorship was 87.8%. Five-year loosening free and acetabular reoperation free survivorships were 80.7% and 81.3%, respectively. They showed that functional outcomes were 28.9, 52.4, 33.7 (WOMAC), and 44.2 those were acceptable.^[21]^ In Paprosky et al study, all 16 of their patients had pelvic discontinuities, the most severe form of deficiency.^[22]^ However, in the report of Rosson and Schatzker,^[23]^ only one of 20 patients had a pelvic deficiency. Not surprisingly, the former study had a much higher revision rate (5 of 16 cages revised within 4.5 years’ average follow-up) than the latter (no revision at 5 years’ average follow-up).

The reported outcomes of acetabular cage reconstruction have been conflicting. Few reports have used actuarial methods in estimating survivorship, thus making comparison of results difficult. The hip joint is formed between the head of the femur and acetabulum which is located outside Innominate bone. The hip joints are one of the Diarthorosis joints (a movable-joint, that is articular surface that lie side by side, can move freely together) and also a ball and socket joint which is a highly developed type of diarthrosis joint and moves in all 3 spatial points.^[24]^ Innominate bone in fetal and childhood until adulthood consists of 3 separate bones called lime, ischium and pubis. The junction of these 3 bones is the acetabulum depth area of the middle area of the acetabulum. Throughout the contact area of these 3 bones, there is a triradiate cartilage and at the ends of each of the 3 categories, there is a growth plate which provides the capacity needed for growth and evolution of these 3 bones.^[25]^ The hip joint is one of biggest and most important joints in the body, there is a hip joint on both sides of the pelvis. The hip joint job is to couple between upper and lower extremity synergies during whole body that plays an important role in maintaining balance. Acetabular fractures are part of hip fractures that is one of the skeletal system injuries. Usually this type of injuries happens as a result of a car accident, a fall from a height or bone loss. Sometimes, as a result of an accident and forces on the femoral head, it hits the acetabulum severely which cause a fracture in and the acetabular cavity. In some cases, it is seen that the effect of the forces applied to pelvis causes tight dislocation of the femoral head, which is now broken. This incident is called a hip joint dislocation.^[26]^ As a consequence of acetabular fractures, the smooth and broken and steady surface of it turned into a broken, uneven surface and no longer is suitable for smooth movement of the femoral head. The vast majority of acetabular fractures can be treated with surgery. The aim of this surgery is to precisely place the fractured parts together until after their union, acetabulum can have a smooth and even surface for femoral to move and slip.^[27]^ After this surgery, bed rest is not needed any more therefore the mentioned problems that caused by long-term rest in bed can be avoided. Severe pain in pelvic is caused by acetabular fractures. It also causes the lower limb movement painful which as a result makes it very hard to stand on foot and in some cases impossible. In order to evaluate the results of utilizing GAP II acetabular cup in acetabulum repairmen in hip revision, some of them have been investigated in this study.

However, the reconstructive procedure is demanding in the absence of a sufficient amount of bone. Such defects have been addressed with the use of structural allograft, highly porous metal shells with or without a cage, or the use of a custom triflange cup.^[28,29]^ In general, structural grafts replacing more than 50% of the acetabulum require protection by a cage. The major advantages of rings and cages are, the cage and ring allow for restoration of hip center; they provide uniform load to the allograft stimulating bone remodeling and incorporation into host bone,^[29]^ allows the surgeon to cement a liner in any position independent of the ring position and the elution of local antibiotics from the cement. The cage protects either the morsellized or structural allograft while it remodels, and if the cage fails cementless revision can be done.^[30]^

Although there are controversial outcomes from GAP II implant for hip reconstruction, we showed in our study low rate of complications with high rate of survival in a nearly large scale population.

## 5. Conclusion

In conclusion, we can say that in order to repair the acetabulum for hip revision, a metal cage should be used to support acetabular and bone graft in reconstructing bone defects. GAP II cage with impaction bone grafting has encouraging midterm results with low failure rate. It could reconstruct severe acetabular bone defects allowing further revision surgeries if needed. GAP II implant has features such as extension plate and a hook for obturator hole. We can say that the utilization of this implant can lead us to desirable results. Our findings showed that using GAP II acetabular cage in the restoration of acetabulum in hip revision surgery is significantly desirable.

## Author contributions

Dr Afshin Taheriazam designed and performed the study. Dr Amin Saeidinia processed the data and analyzed the results. Both of them wrote the first draft and final version.

**Conceptualization:** Afshin Taheriazam.

**Data curation:** Afshin Taheriazam.

**Investigation:** Afshin Taheriazam.

**Methodology:** Afshin Taheriazam, Amin Saeidinia.

**Software:** Amin Saeidinia.

**Writing—original draft:** Amin Saeidinia.

**Writing—review and editing:** Amin Saeidinia.
